# Non-Hermitian Skin Effect in a Non-Hermitian Electrical Circuit

**DOI:** 10.34133/2021/5608038

**Published:** 2021-03-15

**Authors:** Shuo Liu, Ruiwen Shao, Shaojie Ma, Lei Zhang, Oubo You, Haotian Wu, Yuan Jiang Xiang, Tie Jun Cui, Shuang Zhang

**Affiliations:** ^1^School of Physics and Astronomy, University of Birmingham, Birmingham B15 2TT, UK; ^2^State Key Laboratory of Millimeter Waves, Southeast University, Nanjing 210096, China; ^3^School of Physics and Electronics, Hunan University, Changsha 410082, China

## Abstract

The conventional bulk-boundary correspondence directly connects the number of topological edge states in a finite system with the topological invariant in the bulk band structure with periodic boundary condition (PBC). However, recent studies show that this principle fails in certain non-Hermitian systems with broken reciprocity, which stems from the non-Hermitian skin effect (NHSE) in the finite system where most of the eigenstates decay exponentially from the system boundary. In this work, we experimentally demonstrate a 1D non-Hermitian topological circuit with broken reciprocity by utilizing the unidirectional coupling feature of the voltage follower module. The topological edge state is observed at the boundary of an open circuit through an impedance spectra measurement between adjacent circuit nodes. We confirm the inapplicability of the conventional bulk-boundary correspondence by comparing the circuit Laplacian between the periodic boundary condition (PBC) and open boundary condition (OBC). Instead, a recently proposed non-Bloch bulk-boundary condition based on a non-Bloch winding number faithfully predicts the number of topological edge states.

## 1. Introduction

Non-Hermitian systems with gain and loss are very common in the real world [1–5]. The interplay between Non-Hermitian Hamiltonian and topological phases induces many interesting physical phenomena that exhibit substantial differences from their Hermitian counterparts. One of the intriguing recent discoveries is the breakdown of conventional bulk-boundary correspondence [6], a well-known principle used to predict the number of topological edge states of a finite Hermitian system with open boundary condition (OBC) directly from the topological invariant of the same system with periodic boundary condition (PBC) [7]. Yao et al. explained this phenomenon as a result of the non-Hermitian skin effect (NHSE), an exponential decay behavior of eigenstates in non-Hermitian systems with broken reciprocity where most of the eigenstates are localized near the boundary. The NHSE violates the conventional Bloch theorem where all eigenstates extend to the entire bulk with equal intensity [6, 8, 9]. A non-Bloch bulk-boundary correspondence was later established for the non-Hermitian topological systems with a redefinition of a non-Bloch topological invariant in a generalized Brillouin zone [6, 10, 11].

Due to the design flexibility of electrical circuits, it has recently become a powerful platform for studying topological physics and demonstrating some of the topological phenomena, such as topologically protected edge state in the 1-D Su–Schrieffer–Heeger (SSH) model [12, 13], Haldane model and magnetic dipole [14], Weyl state and Fermi arc surface state in three-dimensional(3-D) [15, 16], and higher-order topological states in higher-dimensional circuit lattices [17–19]. The wide range of active devices for electrical circuits has enabled convenient realizations of non-Hermitian topological systems which require precisely controlled gain and/or loss. Operational amplifier (OpAmp) was recently employed as a gain element to realize a reconfigurable non-Hermitian system with a tunable topological bandgap [20], a gain-and-loss induced topological phases [21], and a bulk Fermi-arc state (bulk drumhead states) that connects between the exceptional points (exceptional lines). It is also convenient to introduce strong nonlinear effect into topological circuit by using varactor diodes whose capacitance is dependent on the voltage across it, opening door to realization of self-induced topological states [22, 23] and enhanced harmonic generation in a nonlinear transmission line metamaterial [24].

In this work, we experimentally demonstrate a 1D non-Hermitian topological circuit with broken reciprocity by utilizing a voltage follower module to implement unidirectional coupling. We experimentally observe the topological edge state at the circuit boundary by measuring the impedance spectra between each adjacent node. To confirm the breakdown of conventional bulk-boundary correspondence in such non-Hermitian topological circuits, we compare the differences of phase transition conditions for circuit under the PBC and OBC with both theoretical calculations and numerical simulations. We show that a recently proposed bulk-boundary correspondence in the non-Hermitian regime which introduces a non-Bloch wave vector for the calculation of the topological invariant precisely predicts the number of topological edge mode in such non-Hermitian topological circuits [6].

## 2. Bulk Properties

**Figure 1:**
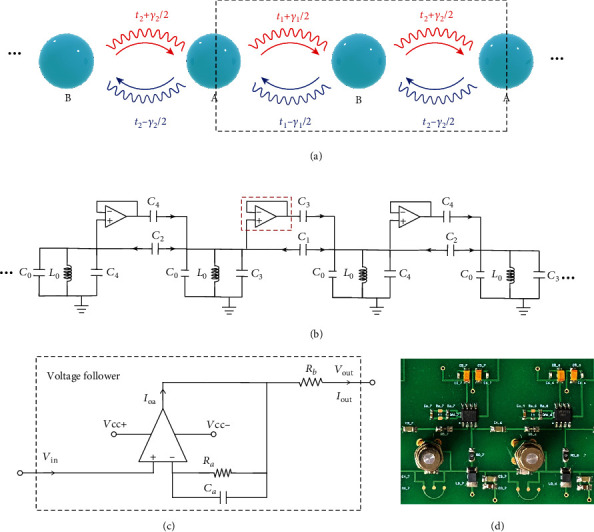
Schematic and circuit diagram of the nonreciprocal non-Hermitian circuit. (a) Schematic of the nonreciprocal non-Hermitian model in the electronic system. The dashed line outlines a single unit cell. (b) Circuit implementation of the nonreciprocal non-Hermitian model. (c) Circuit diagram of the voltage follower module. (d) Real PCB layout of one unit cell of the nonreciprocal non-Hermitian circuit.

We start with the schematic model presented in Figure 1(a), which is a nonreciprocal version of the 1-D Su–Schrieffer–Heeger (SSH) model [12, 13]. Each unit cell comprises of two sites A and B, with nonreciprocal intracell coupling *t*_1_ + *γ*_1_/2 and *t*_1_ − *γ*_1_/2 and nonreciprocal intercell coupling *t*_2_ + *γ*_2_/2 and *t*_2_ − *γ*_2_/2. This model can be physically realized in electrical circuit, as demonstrated in Figure 1(b). Each site is composed of a LC resonant tank with inductance *L*_0_ and capacitance *C*_0_. The nonreciprocal coupling ±*γ*_*i*_/2 is achieved through a one-way coupling capacitor *C*_3_(*C*_4_). This can be realized in electrical circuits in many ways, for example, by connecting *C*_3_(*C*_4_) in series with a voltage follower, as detailed in Figure 1(c), which is composed of an operational amplifier (OpAmp) configured with a negative feedback network. Due to the virtual open and virtual short circuit conditions between the inverting input and noninverting input pins, the current at the left side of the capacitor is blocked, while it remains uninfluenced at the right side, as shown in Figure 1(c). Figure 1(d) shows the photo of a single unit cell of the fabricated sample.

According to Kirchhoff's law, any circuit lattice can be completely described by the admittance matrix (or the circuit Laplacian) as [25]

(1) $$\begin{array}{c} I=\left(i\omega C+1/i\omega W\right)V=J\left(\omega \right)V, \end{array}$$

in which *C* and *W* represent the Laplacian matrices for capacitance and inverse inductance, respectively. Therefore, we can write the circuit Laplacian of the periodic circuit in Figure 1(b) as

(2) $$\begin{array}{c} J\left(\omega ,q\right)=i\omega C+\frac{1}{i\omega }W=i\omega \left[\begin{array}{cc} -\left(C_{0}+C_{1}+C_{2}+C_{3}+C_{4}-1/\omega^{2}L_{0}\right) & C_{1}+C_{3}+C_{2}e^{-iq}\\ C_{1}+\left(C_{2}+C_{4}\right)e^{iq} & -\left(C_{0}+C_{1}+C_{2}+C_{3}+C_{4}-1/\omega^{2}L_{0}\right) \end{array}\right], \end{array}$$

in which *q* is the Bloch wave number relating the voltage between two adjacent unit cells through *V*_*n*±1_(*t*) = *V*_*n*_(*t*) · *e*^±*iq*^. Note that in electrical circuit, because the offdiagonal components (mutual admittance) also contribute to the diagonal component (self-admittance), an additional capacitor *C*_3_/*C*_4_ is connected in parallel with the corresponding site to ensure that all nodes have identical self-admittance.

It is noted that the eigenfrequency of the circuit is obtained from the circuit Hamiltonian which can be constructed in the new basis $\psi \left(t\right)=\left(V\left(t\right),\dot{V}\left(t\right)\right)$ using the circuit Laplacian matrices **C** and **W** (Supplementary Note S1 and Eqs. (S3)-(S4)) [16, 18, 26].

(3) $$\begin{array}{c} H=i\left[\begin{array}{cc} 0 & \frac{W}{C}\\ -I & 0 \end{array}\right]. \end{array}$$

Supplementary Note S1 shows that the circuit Laplacian and circuit Hamiltonian are connected in a way that the zeros of eigenvalue of circuit Laplacian correspond to the eigenvalues of the circuit Hamiltonian (i.e., eigenfrequencies of the circuit).

**Figure 2:**
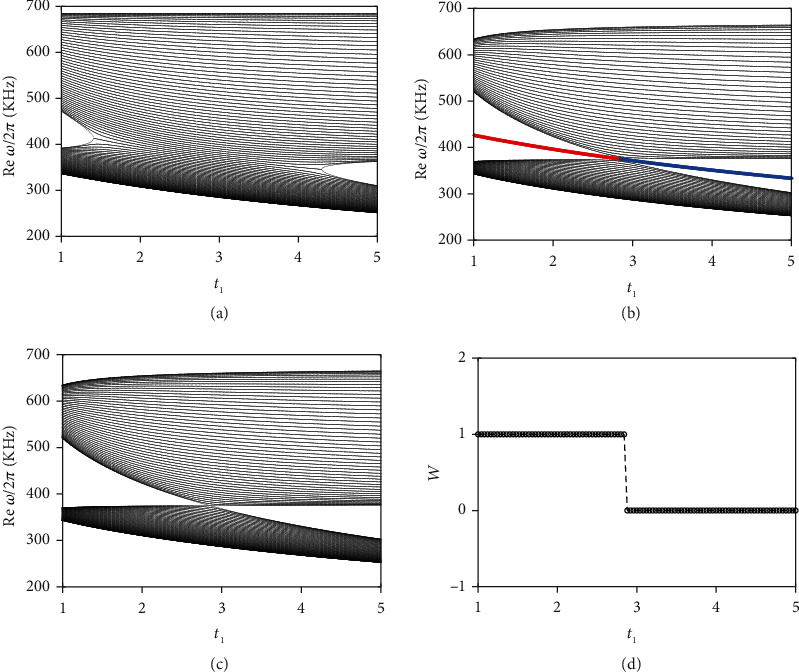
Bulk properties of the of the nonreciprocal non-Hermitian circuit. (a) Bulk band structure as a function of *t*_1_ for fixed *t*_2_ = 2.85 and *γ*_1_ = *γ*_2_ = 1.45, which correspond to real circuit parameters *C*_0_ = 470 pF, *C*_2_ = 1000 pF, *C*_3_ = C_4_ = 680 pF, and *L*_0_ = 47 *μ*H. Each curve in (a) represents an eigenfrequency at certain Bloch momentum *q* in the first Brillouin zone (BZ). (b) The finite band structure as a function of *t*_1_ of a finite circuit chain with 40 unit cells. The circuit parameters are the same as in (a). (c) The bulk band structure as a function of *t*_1_ of the generalized Bloch circuit Hamiltonian. Each curve in (c) represents an eigenfrequency at certain non-Bloch wavevector *β* in the first BZ. (d) Non-Bloch winding number calculated using the generalized Bloch circuit Hamiltonian in the generalized Brillouin zone with the same circuit parameters in Figure 2(a).

Note that *ω* in the circuit Laplacian Eq. (2) is not the eigenfrequency but an external parameter. For $\omega_{0}=1/\sqrt{\left(C_{0}+C_{1}+C_{2}+C_{3}+C_{4}\right)L_{0}}$, the diagonal components of *J*(*ω*_0_, *q*) vanishes, and Eq. (2) simplifies to the block offdiagonal form

(4) $$\begin{array}{c} J\left(\omega_{0},q\right)=i\omega_{0}C_{0}\left\{\left[d_{x}+i\gamma_{2}/2\sin\left(q\right)\right]\sigma_{x}+\left[d_{y}+i\left(\gamma_{1}/2-\gamma_{2}/2\cos\left(q\right)\right)\right]\sigma_{y}\right\}, \end{array}$$

where *t*_1_ = 2*C*_1_ + *C*_3_/2*C*_0_, *t*_2_ = 2*C*_2_ + *C*_4_/2*C*_0_, *γ*_1_ = *C*_3_/*C*_0_, *γ*_2_ = *C*_4_/*C*_0_, *d*_*x*_ = *t*_1_ + *t*_2_cos(*q*), *d*_*y*_ = *t*_2_sin(*q*), and *σ*_*x*,*y*_ are the Pauli matrices. Due to the chiral symmetry *σ*_*z*_^−1^**J**(*ω*_0_, *q*)*σ*_*z*_ = −**J**(*ω*_0_, *q*), the eigenvalues of **J**(*ω*_0_, *q*) come in pairs, given by$j_{\pm }=\pm i\omega_{0}C_{0}\sqrt{{\left[d_{x}+i\gamma_{2}/2\cdot \sin\left(q\right)\right]}^{2}+{\left[d_{y}+i\left(\gamma_{1}/2-\gamma_{2}/2\cdot \cos\left(q\right)\right)\right]}^{2}}$. The gap closing condition (*t*_1_ − *t*_2_)^2^ − (*γ*_1_/2 + *γ*_2_/2)^2^ = 0 can be found by letting *j*_±_ = 0, which is achieved only at *q* = ±*π*. This is confirmed by the band structures of the bulk circuit at three different *t*_1_ of 1.2, 2.85, and 4.8, as detailed in Supplementary Figure S1a. We first consider the special case *γ*_1_ = *γ*_2_ = *γ*, which leads to the phase transition condition *t*_1_ = *t*_2_ ± *γ*.  Figure 2(a) shows the bulk eigenfrequencies as a function of *t*_1_ for fixed *t*_2_ = 2.85 and *γ*_1_ = *γ*_2_ = 1.45, which correspond to circuit parameters *C*_0_ = 470 pF, *C*_2_ = 1000 pF, *C*_3_ = C_4_ = 680 pF, and *L*_0_ = 47 *μ*H. Each curve in Figure 2(a) represents an eigenfrequency at certain Bloch momentum *q* in the first Brillouin zone (BZ). Two transition points at *t*_1_ = 1.4 and 4.3 are clearly observed in Figure 2(a), which coincide exactly with the theoretical value *t*_1_ = *t*_2_ ± *γ*. It is noted that the gap closing condition derived at frequency *ω*_0_ is valid for the entire frequency spectrum, due to the continuity of the eigenvalue *j_n_* of circuit Laplacian.

In comparison, for a finite circuit chain with *N* = 40 unit cells, the two transition points merge into a single one at *t*_1_ = *t*_2_ = 2.85, as shown in Figure 2(b). An isolated curve observed in the bulk band gap represents the topological edge state. One may notice that the edge mode exists on both sides of the transition points, which is due to the difference of boundary termination between electrical circuits and quantum systems, as is clarified by Supplementary Figure S2. The mid gap mode on the left (red curve) and right (blue curve) side of the transition points represents the edge mode at the right and left boundary, respectively, which are confirmed in Supplementary Figure S3 for the eigenstates of the left and right edge modes. We explain the discrepancy of the band structure between PBC and OBC by considering a finite circuit Laplacian **J**(*ω*_0_). [6] Let us construct a 2 *N* × 2 *N* diagonal matrix **S** = diag(1, r_1_^1^, r_1_^1^r_2_^1^, r_1_^2^r_2_^1^, r_1_^2^r_2_^2^, ⋯, r_1_^*N*−1^r_2_^*N*−2^, r_1_^*N*−1^r_2_^*N*−1^, r_1_^*N*^r_2_^*N*−1^), in which the general form of the odd and even diagonal components are [*S*]_*odd*,*n*_ = *r*_1_^*n*−1/2^*r*_2_^*n*−1/2^ and [*S*]_even,*n*_ = *r*_1_^*n*/2^*r*_2_^*n*/2−1^, respectively, with $r_{1}=\sqrt{\left|\left(t_{1}-\gamma_{1}/2\right)/\left(t_{1}+\gamma_{1}/2\right)\right|}$ and $r_{2}=\sqrt{\left|\left(t_{2}-\gamma_{2}/2\right)/\left(t_{2}+\gamma_{2}/2\right)\right|}$. Then, we can transforms the nonreciprocal circuit Laplacian **J**(*ω*_0_) into a reciprocal one $\bar{J}\left(\omega_{0}\right)=SJ\left(\omega_{0}\right)S^{-1}$, which is equivalent to the standard reciprocal SSH model with intracell and intercell couplings ${\bar{t}}_{1}=\sqrt{\left(t_{1}+\gamma_{1}/2\right)\left(t_{1}-\gamma_{1}/2\right)}$ and ${\bar{t}}_{2}=\sqrt{\left(t_{2}+\gamma_{2}/2\right)\left(t_{2}-\gamma_{2}/2\right)}$, respectively. Obviously, the gap closing condition for the OBC becomes ${\bar{t}}_{1}={\bar{t}}_{2}$, which results in the phase transition point at $t_{1}=\pm \sqrt{t_{2}^{2}+{\left(\gamma_{1}/2\right)}^{2}-{\left(\gamma_{2}/2\right)}^{2}}$. For *γ*_1_ = ±*γ*_2_, we have *t*_1_ = ±*t*_2_, which perfectly matches with the numerical results in Figure 2(b). Noted that in spite of the differences between the circuit Hamiltonian and circuit Laplacian, they share the same transition points, as are confirmed by the eigenvalue spectra of **J**(*ω*_0_, *q*) provided in Supplementary Figure S1b and c. The pronounced deviations observed in the band structure from the PBC (Figure 2(a)) to OBC (Figure 2(b)) imply that the bulk band structure no longer describes a finite system with open boundaries in such non-Hermitian systems. The bulk transition point for the case *γ*_1_ = ‐*γ*_2_ = *γ* becomes *t*_1_ = *t*_2_, which happens to coincide with that of the finite chain, as illustrated in Supplementary Figure S4.

Several new topological invariants have been defined to characterize the non-Hermitian systems, including the Chern number [27–29], generalized Berry phase [30–32], and winding numbers [6, 10, 11, 33–37]. Here, we employ the non-Bloch winding number as the topological invariant [6], which is calculated based on a non-Bloch circuit Laplacian. Inspired by the phenomenon of NHSE that all eigenstates are tightly confined to the system boundary, the conventional Bloch wave number *e*^*iq*^ should be replaced by a non-Bloch wave number *β* = *re*^*iq*^. Here, *r* = *r*_1_*r*_2_ is a real positive number representing the level of eigenstate localization. The generalized Brillouin zone with the same circuit parameters used in the experiment is presented in Supplementary Material Figure S5. Thus, the non-Bloch circuit Laplacian is

(5) $$\begin{array}{c} \tilde{J}\left(\omega_{0},\beta \right)={\tilde{U}}_{+}\left(\omega_{0},\beta \right)\sigma_{+}+{\tilde{U}}_{-}\left(\omega_{0},\beta \right)\sigma_{-}, \end{array}$$

in which, ${\tilde{U}}_{+}\left(\omega_{0},\beta \right)=i\omega_{0}\left(C_{1}+C_{3}+C_{2}r_{1}^{-1}r_{2}^{-1}e^{-iq}\right)$, ${\tilde{U}}_{-}\left(\omega_{0},\beta \right)=i\omega_{0}\left(C_{1}+\left(C_{2}+C_{4}\right)r_{1}r_{2}e^{iq}\right)$, and *σ*_±_ = (*σ*_*x*_ + *iσ*_*y*_)/2. The bulk band structure of the non-Bloch circuit Hamiltonian $\tilde{H}\left(\beta \right)$ is shown in Figure 2(c), which exactly coincides with that of the open circuit (Figure 2(b)). To calculate the topological invariant of $\tilde{J}\left(\omega_{0},\beta \right)$, a generalization of the usual “Q matrix” is defined as [38]

(6) $$\begin{array}{c} Q=|{\tilde{u}}_{R,+}\rangle \langle {\tilde{u}}_{\text{L},+}|-|{\tilde{u}}_{R,-}\rangle \langle {\tilde{u}}_{\text{L},-}|=\frac{1}{\sqrt{{\tilde{U}}_{+}{\tilde{U}}_{-}}}\left(\begin{array}{cc} 0 & {\tilde{U}}_{+}\\ {\tilde{U}}_{-} & 0 \end{array}\right), \end{array}$$

in which $|{\tilde{u}}_{R,\pm }\rangle$ and $|{\tilde{u}}_{\text{L},\pm }\rangle$ are the right and left eigenstates satisfying $\langle {\tilde{u}}_{\text{L},\pm }|{\tilde{u}}_{R,\pm }\rangle =\delta_{+-}$

(7) $$\begin{array}{c} |{\tilde{u}}_{R,\pm }\rangle =\frac{1}{\sqrt{2{\tilde{U}}_{+}{\tilde{U}}_{-}}}\left(\begin{array}{c} {\tilde{U}}_{+}\\ \pm \sqrt{{\tilde{U}}_{+}{\tilde{U}}_{-}} \end{array}\right),\\ |{\tilde{u}}_{\text{L},\pm }\rangle =\frac{1}{\sqrt{2{\tilde{U}}_{+}^{†}{\tilde{U}}_{-}^{†}}}\left(\begin{array}{c} {\tilde{U}}_{-}^{†}\\ \pm \sqrt{{\tilde{U}}_{+}^{†}{\tilde{U}}_{-}^{†}} \end{array}\right). \end{array}$$

The non-Bloch winding number is defined as

(8) $$\begin{array}{c} W=\frac{i}{2\pi }\int_{C_{\beta }}g^{-1}dg, \end{array}$$

in which

(9) $$\begin{array}{c} g=\frac{{\tilde{U}}_{+}}{\sqrt{{\tilde{U}}_{+}{\tilde{U}}_{-}}},g^{-1}=\frac{{\tilde{U}}_{-}}{\sqrt{{\tilde{U}}_{+}{\tilde{U}}_{-}}}. \end{array}$$

The integration is proceeded along a nonunit circle *β* = *re*^*iq*^, which becomes a unit circle for the Hermitian case.  Figure 2(d) shows the numerical results of the non-Bloch winding number *W*, which jumps from 1 to 0 at *t*_1_ = *t*_2_ = 2.85. As mentioned above, due to the boundary termination of our circuit, *W* = 1 and *W* = 0 indicate the appearance of topological edge states at the left and right ends of the circuit, respectively. In this regard, we have recovered the bulk-boundary correspondence in non-Hermitian topological circuit, named as non-Bloch correspondence.

## 3. Topological Edge Stage in the Finite Circuit

The nontrivial topological feature predicted from the finite band structure (Figure 2(b)) of our nonreciprocal topological circuit is indicated by a prominent edge state confined at the circuit boundaries. In both quantum and photonics systems, topological edge states can be directly measured by the state intensity on each node. However, as have been mentioned in many previous literatures, the topological edge states in electrical circuits are manifested by a strong resonant peak in the impedance spectra measured between two adjacent nodes [13, 15, 21, 39].

**Figure 3:**
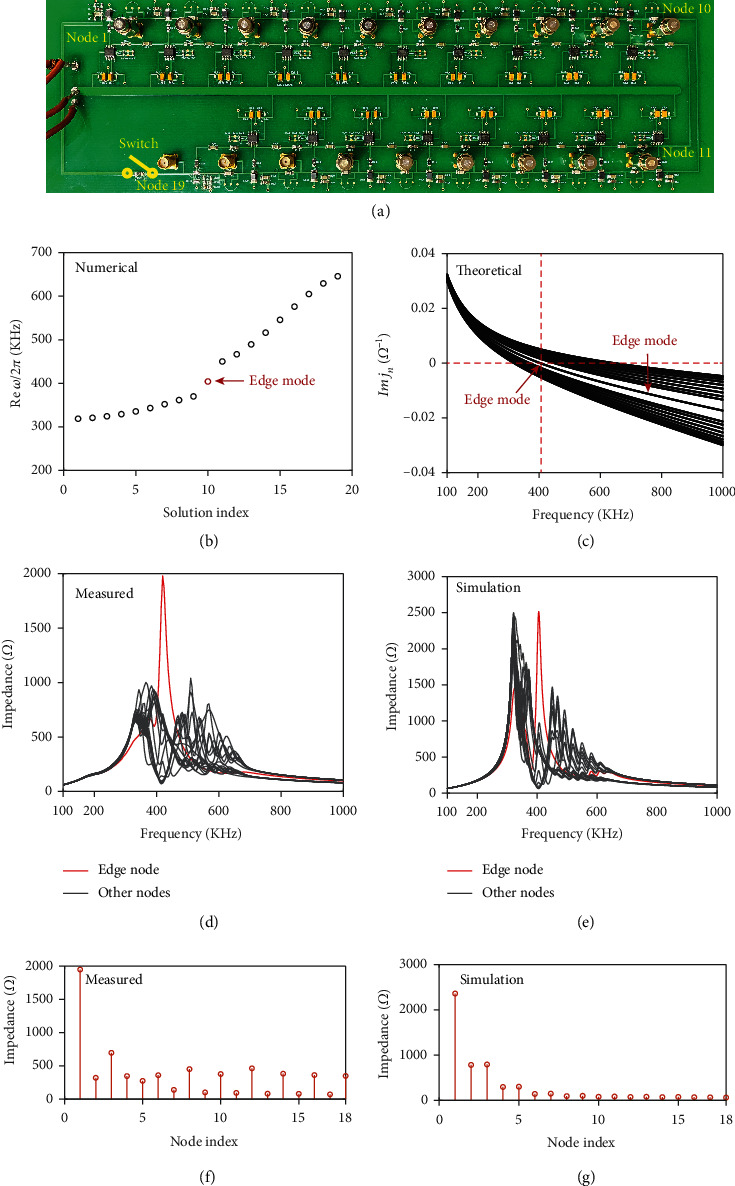
Experimental and numerical results for the topological edge mode. (a) Fabricated sample of the finite circuit chain containing 9.5 unit cells (19 nodes), with circuit parameters *C*_0_ = 470 pF, *C*_1_ = 470 pF, *C*_2_ = 1000 pF, *C*_2_ = 1000 pF, *C*_3_ = C_4_ = 680 pF, and *L*_0_ = 47 *μ*H, corresponding to*t*_1_ = 1.72, *t*_2_ = 2.85, and *γ*_1_ = *γ*_2_ = 1.45. (b) Sorted eigenfrequencies of the finite circuit chain. Red circle represents the topological edge mode. (c) Imaginary part of the eigenvalue spectra of *J*(*ω*) for the finite circuit chain. The isolated curve represents the edge mode. (d, e) Experimentally measured and numerically calculated impedance spectra of the finite circuit chain, respectively. Red curve indicates the impedance measured across the leftmost coupling capacitors. (f, g) Experimentally measured and numerically calculated impedance distributions of the finite circuit chain. Note that the impedance spectra in all experiments and simulations are measured across the each adjacent node (i.e., across each coupling capacitor).

To experimentally observe the topological edge state in our nonreciprocal electrical circuit, a sample containing 9.5 unit cells (19 nodes in total) is fabricated, as shown in Figure 3(a). A 0 *Ω* resistor pad is designed between node 1 and node 19 to allow the circuit to switch between the OBC and PBC scenarios. The circuit parameters are chosen to be the same as those in Figure 2(a), which are *C*_0_ = 470 pF, *C*_1_ = 470 pF, *C*_2_ = 1000 pF, *C*_2_ = 1000 pF, *C*_3_ = C_4_ = 680 pF, and *L*_0_ = 47 *μ*H, corresponding to*t*_1_ = 1.72, *t*_2_ = 2.85, and *γ*_1_ = *γ*_2_ = 1.45.

First, we investigate the finite circuit chain with OBC by turning off the switch. Figure 3(b) shows the real part of the calculated eigenfrequency spectra of the finite circuit. A mid-gap eigenmode (red circle) at around 404 KHz represents the topological edge state confined at the left boundary of the circuit. The edge mode is also indicated in the imaginary part of eigenvalues of the finite circuit Laplacian (Figure 3(c)) by an isolated curve, which crosses zero at exactly the frequency of edge state (404 KHz), while the remaining curves represent all the bulk modes. The measured and simulated impedance spectra between all adjacent nodes are shown in Figures 3(d) and 3(e), respectively. A distinct peak (red curve) of nearly 2000 *Ω* representing the topological edge state between leftmost nodes is clearly identified at 404KHz, far exceeding the impedance between all the other adjacent nodes (black curves) which represent the bulk mode. Figures 3(f) and 3(g) further provide the impedance distributions at the edge mode frequency for the measured and simulation results, respectively, where the topological edge state is clearly identified at the left edge and quickly decays into the bulk. Discrepancies observed in the impedance spectra between the simulation and experimental results are due to the real working status of the OpAmp in the experiment, which is sensitive to the peripheral circuit and is easily affected by many factors in experiment.

**Figure 4:**
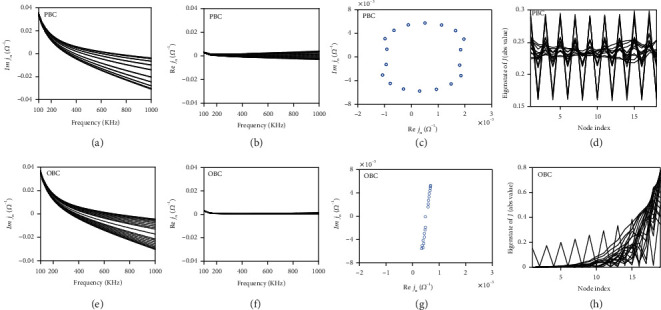
Experimental results for the eigenvalue and eigenstates of the finite circuit Laplacian under PBC and OBC. (a, b) The imaginary and real part of eigenvalues of the closed-loop circuit Laplacian under PBC, respectively. (c, d) The eigenvalues *j*_*n*_(*ω*_0_) and their eigenstates of the circuit Laplacian at the mid-gap frequency *ω*_0_ for the finite circuit under PBC. (e, f) The imaginary and real part of eigenvalues of the closed-loop circuit Laplacian under OBC, respectively. (g, h) The eigenvalues *j_n_*(*ω*_0_) and their eigenstates of the circuit Laplacian at the mid gap frequency *ω*_0_ for the finite circuit under OBC. Note that the eigenstates are plotted in absolute values.

Next, we investigate how the above circuit behaves under the PBC and OBC by turning on and off the switch, respectively. To ensure a strict PBC with a total number of 9 unit cells (18 nodes), node 19 is removed, and the grounding term at node 1 is adjusted to fit the PBC illustrated in Figure 1(b). The circuit Laplacian of the periodic circuit is experimentally obtained by firstly measuring the *N*-port *S*-parameter of the entire network (18 nodes) and then transforming it into circuit Laplacian (admittance matrix, see method). Figures 4(a) and 4(b) show the experimental results of the imaginary part and real part of eigenvalue spectra *j*_*n*_(*ω*) of the closed-loop circuit Laplacian, respectively, while Figures 4(e) and 4(f) present the experimental results of the imaginary part and real part of eigenvalue spectra *j*_*n*_(*ω*) of the open-loop circuit Laplacian, respectively. The experimental results shown in both cases are highly consistent with the numerical results given in supplementary Figure S6 a,b and Figure S7a,b. The absence of the isolated curve in imaginary part of the eigenvalue spectra *j*_*n*_(*ω*) in the PBC case (Figure 4(a)) implies the vanishing of topological edge state in the closed-loop circuit. The dramatic change of the circuit Laplacian from PBC to OBC can be more obviously noticed from the complex eigenvalues *j*_*n*_(*ω*_0_) at the mid-gap frequency *ω*_0_, as are illustrated in Figures 4(c) and 4(g) for the PBC and OBC cases, respectively. The eigenvalues *j*_*n*_(*ω*_0_) for the PBC case take nonzero values in both real and imaginary parts, forming an almost equally spaced loop in the complex plane. In sharp contrast, the eigenvalues *j*_*n*_(*ω*_0_) for the OBC case collapse into a gapped line with nearly identical real parts. The isolated circle located inside the gap with zero imaginary parts represents the topological edge mode. The measured eigenvalues *j*_*n*_(*ω*_0_) (Figures 4(c) and 4(g)) are in excellent agreement with the numerical results (Supplementary Figure S6c, Figure S7c).

The difference between the PBC and OBC of the nonreciprocal circuit lattice is also manifested by their eigenstates, as shown in Figures 4(d) and 4(h), respectively. For the PBC case (Figure 4(d)), all the measured eigenstates of **J**(*ω*_0_) oscillate in the entire chain with almost identical intensity, which agrees reasonably well with the simulation results (Supplementary Figure S6d). However, we see from Figure 4(h) that all eigenstates of the open chain (OBC) are exponentially localized at the end of the chain, which again matches well with the simulation results (Supplementary Figure S7d) and indicates the non-Hermitian skin effect [21, 31, 35].

Now, we discuss how the nonreciprocal factor *r*_1_/*r*_2_ affects the localization of eigenstates (NHSE) of **J**(*ω*_0_) in the open circuit. For *r*_1_*r*_2_ > 1, all the eigenstates are localized at the left end of the chain (Supplementary Figure S8c), while for *r*_1_*r*_2_ < 1, all the eigenstates are localized at the right end of the chain (Supplementary Figure S8d). The value of *r*_1_*r*_2_ also determines the level of localization; that is, the larger the |log(*r*_1_*r*_2_)| is, the more likely the eigenstates will be confined to the circuit boundaries. See more details in Supplementary Note S2. Note that during the submission of this manuscript, we noticed another work reported on the experimental observation of the NHSE [40]. However, there are substantial differences between our work and theirs. There are two independent nonreciprocal factors *r*_1_ and *r*_2_ controlling the intracell and intercell coupling in our model, while the nonreciprocal effect only exists in the intracell coupling in the model reported in Ref. 40. The additional nonreciprocal coupling provides us not only higher flexibility in manipulating the level NHSE but also a more generalized form of the nonreciprocal non-Hermitian topological circuit.

## 4. Conclusion

To conclude, we have proposed and experimentally demonstrated a 1-D non-Hermitian topological circuit with nonreciprocal couplings. We analytically derive the phase transition conditions for the circuit under PBC and OBC, which perfectly match with experimental measurements and numerical simulations. A non-Bloch Hamiltonian with *β* = *re*^*iq*^ as the non-Bloch wave number is defined which allows the calculation of topological invariant in the generalized Brillouin zone and extends the bulk-boundary correspondence to non-Hermitian systems [6]. Topological circuit provides us with a convenient platform for experimentally demonstrating new topological physics. The highly flexible configurations OpAmp allow a wide range of circuit functions, including addition, subtraction, integration, and differential, to be applied to the design of hopping parameters of topological circuits. The nonlinear effect can also be conveniently introduced to the non-Hermitian topological circuit by using nonlinear circuit elements, such as transistors and varactor diodes [22, 24, 25]. Our work highlights the interesting features of non-Hermitian topological circuit with nonreciprocity and suggests a convenient experimental platform for future investigation of non-Hermitian topological physics.

## 5. Method

A voltage feedback operational amplifier (Taxes Instrument, LM6171) was used in the experiment to construct the voltage follower module, which blocks the input current while keeping the output current same as in the case without the voltage follower. High-Q inductors (Murata, Q-factor>40, 2% tolerance) were used in experiments to ensure sharp impedance resonance and ideal circuit performance. Additional circuit elements are added in the real circuit to guarantee the stability of the OpAmp, including a *R*_*b*_ = 5 *Ω* resistor connecting in series in each *C*_3_/*C*_4_ branch, a resistor *R*_*a*_ = 2000 *Ω* in shunt with a capacitor *C*_*a*_ = 100 pF connecting across the inverting input and output of OpAmp. In measurement, a pair of filter capacitors (2.2 *μ*F and 2 pF) were connected in parallel with the output of DC supply (Agilent E3648A) to provide ±15 V DC voltage for the OpAmp with minimized ripple current. The impedance spectra of the circuit were measured by a vector network analyzer (VNA, Tektronix TTr500) via the preset SMA ports between adjacent nodes. The PSpice model for the OpAmp officially provided by Texas Instruments was employed in the circuit simulation to deliver accurate numerical results. The measurement of the *N*-port *S*-parameter was achieved by measuring the two-port *S*-parameter for every combination of the 18 nodes using the two-Port VNA. In each measurement, the rest of the ports were connected with 50 *Ω* load terminator. The *S*-parameter matrix *S* was then transformed into admittance matrix (circuit Laplacian) using **Y** = (**I** + **S**)^−1^(**I** − **S**)/*Z*_0_, in which **I** is the identity matrix, and *Z*_0_ is the port impedance of VNA.

## Data Availability

Supplementary materials contain additional data needed to evaluate the conclusions of the paper. All other data used to support the findings of this study are available from the corresponding author upon request.

## Supplementary Materials

Supplementary Materials Figure S1: band structure and eigenvalue spectra of circuit Laplacian under the PBC and OBC. Figure S2: two different types of boundary termination for an SSH-type topological circuit. Figure S3: eigenstates of the circuit Hamiltonian for the finite circuit investigated in Figure 2(b) in the main context. Figure S4: band structures for the case *γ*_1_ = ‐*γ*_2_ = *γ*, with phase transition condition *t*_1_ = *t*_2_ for both PBC and OBC. Supplementary Figure S5: plot of the generalized Brillouin zone with the circuit parameters used in the experiment. Figure S6: numerical results for the eigenvalue and eigenstates of the finite circuit Laplacian for the finite circuit with PBC. Figure S7: numerical results for the eigenvalue and eigenstates of the finite circuit Laplacian for the finite circuit with OBC. Figure S8: band structures and localization of eigenstates in the open circuit chain with *r*_1_*r*_2_ > 1 and *r*_1_*r*_2_ < 1.
