# Supplementary material for: Non-Hermitian Skin Effect in a Non-Hermitian Electrical Circuit
Source: Research (Wash D C). 2021 Mar 15;2021:5608038. doi: 10.34133/2021/5608038 (PMC7989004; doi:10.34133/2021/5608038)
Supplement: Supplementary Materials — Figure S1: band structure and eigenvalue spectra of circuit Laplacian under the PBC and OBC. Figure S2: two different types of boundary termination for an SSH-type topological circuit. Figure S3: eigenstates of the circuit Hamiltonian for the finite circuit investigated in Figure 2(b) in the main context. Figure S4: band structures for the case γ1 = ‐γ2 = γ, with phase transition condition t1 = t2 for both PBC and OBC. Supplementary Figure S5: plot of the generalized Brillouin zone with the circuit parameters used in the experiment. Figure S6: numerical results for the eigenvalue and eigenstates of the finite circuit Laplacian for the finite circuit with PBC. Figure S7: numerical results for the eigenvalue and eigenstates of the finite circuit Laplacian for the finite circuit with OBC. Figure S8: band structures and localization of eigenstates in the open circuit chain with r1r2 > 1 and r1r2 < 1. [file 5608038.f1.docx]

**Supplementary Materials for**

**Non-Hermitian skin effect in a non-Hermitian electrical circuit**

Shuo Liu^1,†^, Ruiwen Shao^2,†^, Shaojie Ma^1,†^, Lei Zhang^2^, Oubo You^1^, Haotian Wu^2^, Yuan Jiang Xiang^3,*^, Tie Jun Cui^2,*^, Shuang Zhang^1,*^

^1^School of Physics and Astronomy, University of Birmingham, Birmingham B15 2TT, United Kingdom

^2^ State Key Laboratory of Millimeter Waves, Southeast University, Nanjing 210096, China

^3^ School of Physics and Electronics, Hunan University, Changsha 410082, China

†These authors contributed equally to this work

*Corresponding author

Corresponding authors: S. Zhang: [S.Zhang@bham.ac.uk](mailto:S.Zhang@bham.ac.uk); T. J. Cui: [Tjcui@seu.edu.cn](mailto:Tjcui@seu.edu.cn); Y. J. Xiang: Xiang78@hnu.edu.cn;

**This PDF file includes:**

Supplementary Text Note.1 to Note 2

Supplementary Figures S1 to S6

**Note S1. Derivation of circuit Hamiltonian**

Different from the Hamiltonian in electronics and photonic systems which directly gives the eigenfrequency of the system, the circuit Laplacian itself is dependent on frequency, thus, the eigenvalues of ***J****(ω,q)* do not correspond to the eigenfrequency of the system. There are two different methods for calculating the eigenfrequencies of a dissipative circuit from the circuit Laplacian. The first method is to solve the roots of the determinant of ***J****(ω,q)* (*det(****J****(ω,q))=0*) [1], which may result in considerable computational burden, and become impossible to solve when the dimension of matrix ***J****(ω,q)* exceeds five.

The second approach is to construct circuit Hamiltonian from the circuit Laplacian ***J****(ω,q)* based on matrices ***C****,* ***W***. Now we briefly introduce how to obtain the circuit Hamiltonian for a topological circuit. If we set *I_a_*=0 in the equation of motion in Eq. (1) in the main context, the evolution of circuit is then determined only by its eigenfrequencies. Define a new basis *ψ(t)* that is comprised of voltage $V(\mathrm{t})$ and its first derivative ,

(S1)

As ***C***, ***W***, and ***J****(ω,q)* are *N×N* matrices for an *N*-node circuit network, Eq. (S1) can hence be rewritten into *2N* differential equations,

(S2)

where the *2N×2N* circuit Hamiltonian takes the following form, in which matrices ***C****,* ***W****,* ***σ*** are given in Supplementary Eqs. (S3)-(S4).

$C=\left[\begin{matrix} -\left(C_{0}+C_{1}+C_{2}+C_{3}+C_{4}\right) & C_{1}+C_{3}+C_{2}e^{-iq} \\ C_{1}+\left(C_{2}+C_{4}\right)e^{iq} & -\left(C_{0}+C_{1}+C_{2}+C_{3}+C_{4}\right) \end{matrix}\right]$ (S3)

$W=\left[\begin{matrix} -\left(1/L_{0}\right) & 0 \\ 0 & -\left(1/L_{0}\right) \end{matrix}\right]$ (S4)

This method consumes much less computational resource and can also give the eigenstate of the system in the basis of .There is a connection between the spectra of the finite Hamiltonian and the eigenvalues of the finite circuit Laplacian. For example, the emergent midgap eigenmode corresponds to an isolated curve in the plot of eigenvalues of ***J****(ω)* for all *ω*, which crosses zero at exactly the frequency of midgap mode.


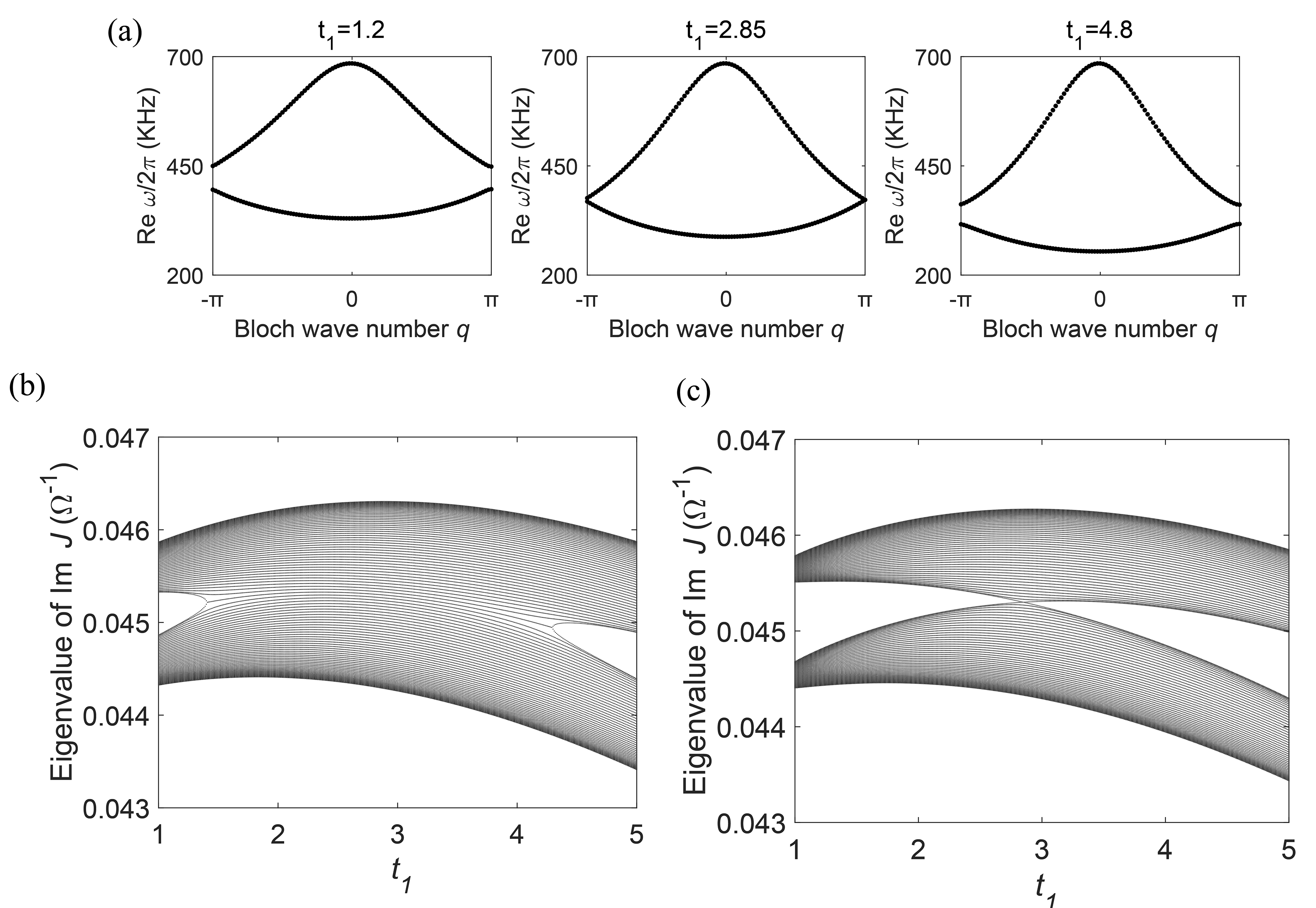


**Supplementary Figure S1. Band structure and eigenvalue spectra of circuit Laplacian under the PBC and OBC. (a)** Band structures of the bulk circuit at three different *t_1_* =1.2, 2.85, 4.8. (b) Simulated eigenvalue spectra of$J\left(\omega_{0},q\right)$as a function of *t_1_*. The circuit parameters are the same with those set for Figure 2a in the main context. (c) Simulated eigenvalue spectra of$J\left(\omega_{0},q\right)$as a function of *t_1_*. The circuit parameters are the same with those set for Figure 2c in the main context. Note that to provide a clear view of the transition points in (b) and (c), the presented data in both graphs has been subtracted by 0.0035*t_1_*.

Now we explain why topological edge mode exists on bode sides of the transition point in Figure 2b in the main context. Let us consider an SSH-type topological circuit with two different boundary terminations, as illustration in Supplementary Figure S2. Type-A (Figure S2a): For C_1_<C_2_, topological edge state appears on the left boundary; For C_1_>C_2_, topological edge state appears on the right boundary. This is because the circuit with type-A boundary termination respects C2 symmetry as we exchange the value of C_1_ and C_2_. Type-B (Figure S2b): For C_1_<C_2_, topological edge state appears on both ends of the circuit; For C_1_>C_2_, topological edge state appears on the right boundary; For C_1_>C_2_, no topological edge states. Note that Type-A boundary termination includes half integer number of unit cell and is not allowed in the electronic model.


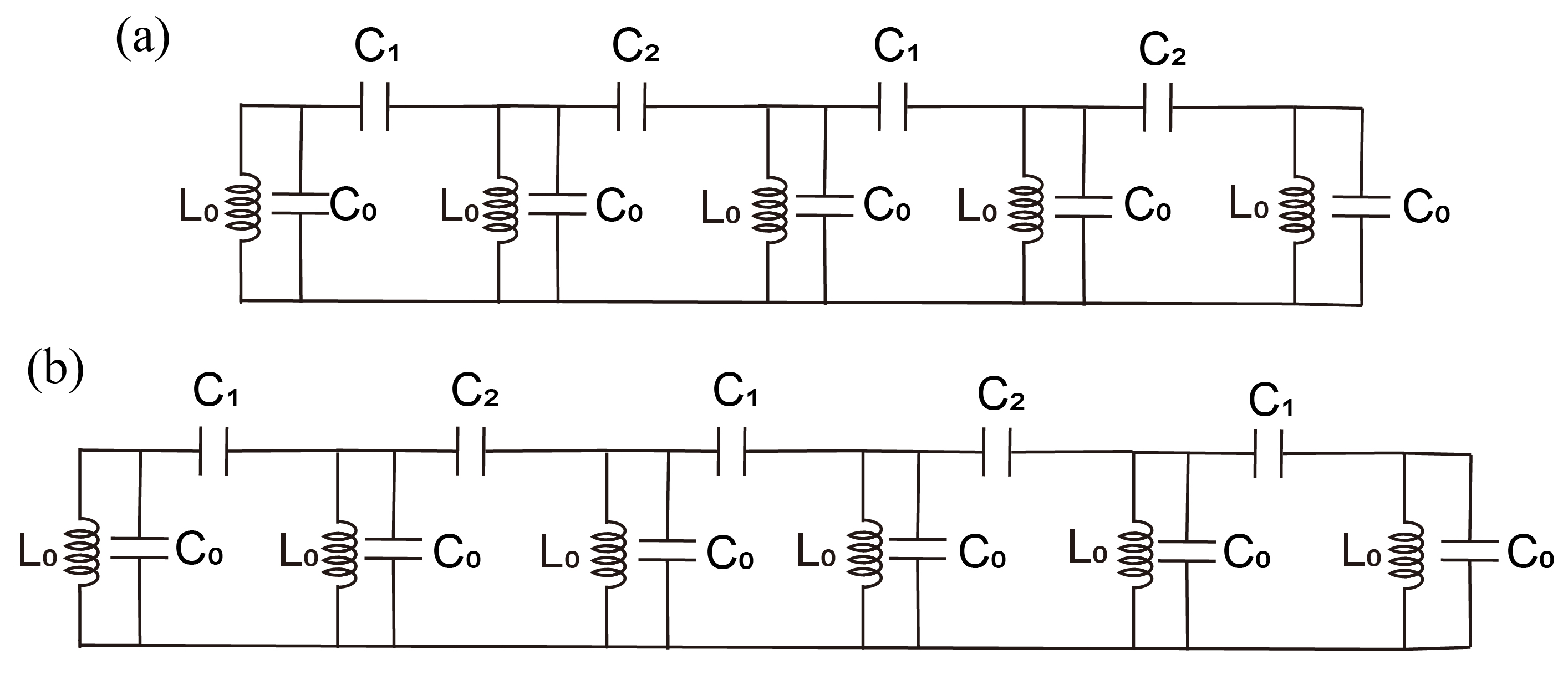


**Supplementary Figure S2. Two different types of boundary termination for an SSH-type topological circuit. (a)** Type-A **(b)** Type-B.


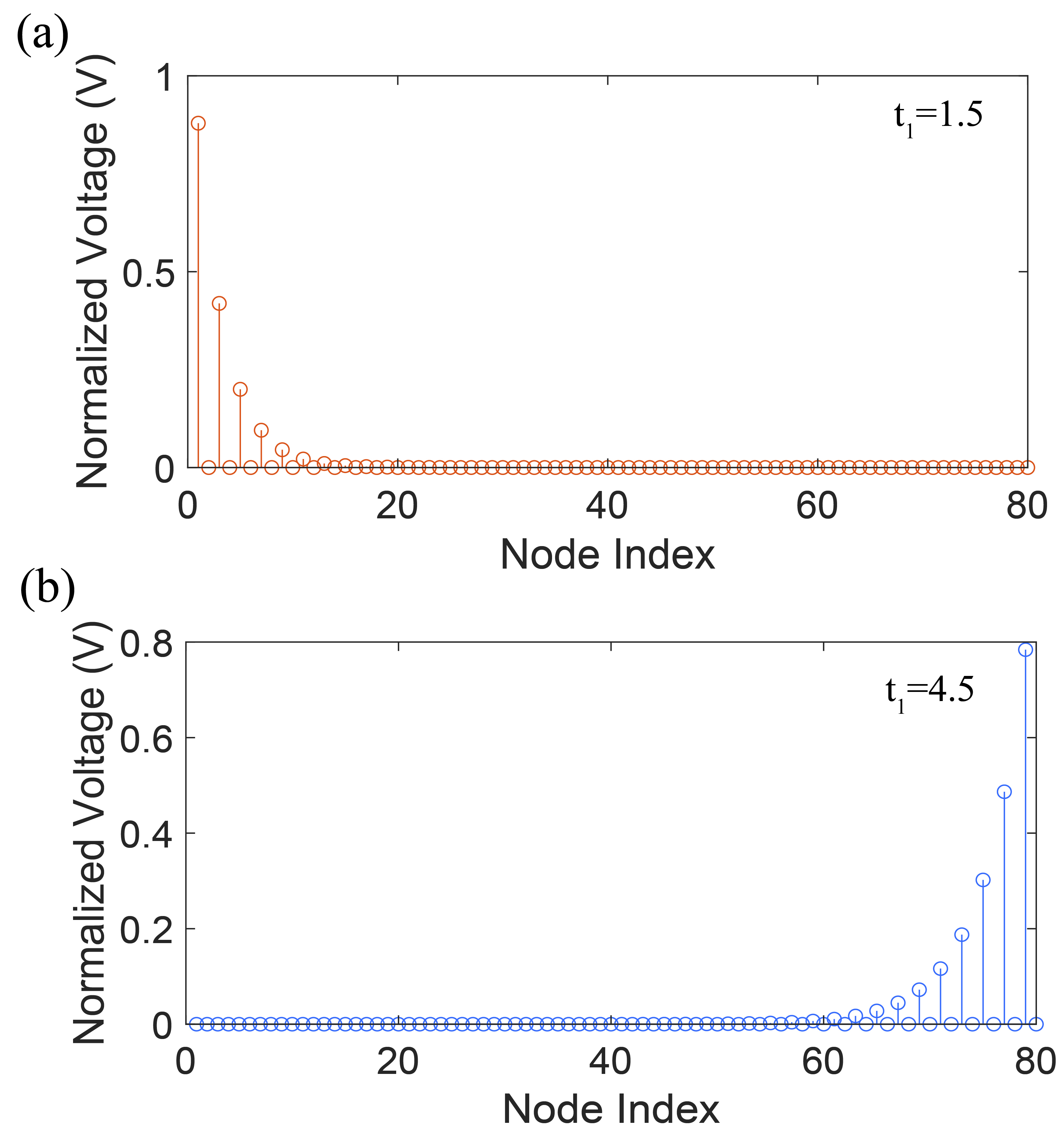


**Supplementary Figure S3. Eigenstates of the circuit Hamiltonian for the finite circuit investigated in Figure 2b in the main context. (a)** For *t_1_*=1.5, the topological edge appears at the left boundary of the circuit. **(b)** For *t_1_*=4.5, the topological edge appears at the right boundary of the circuit.


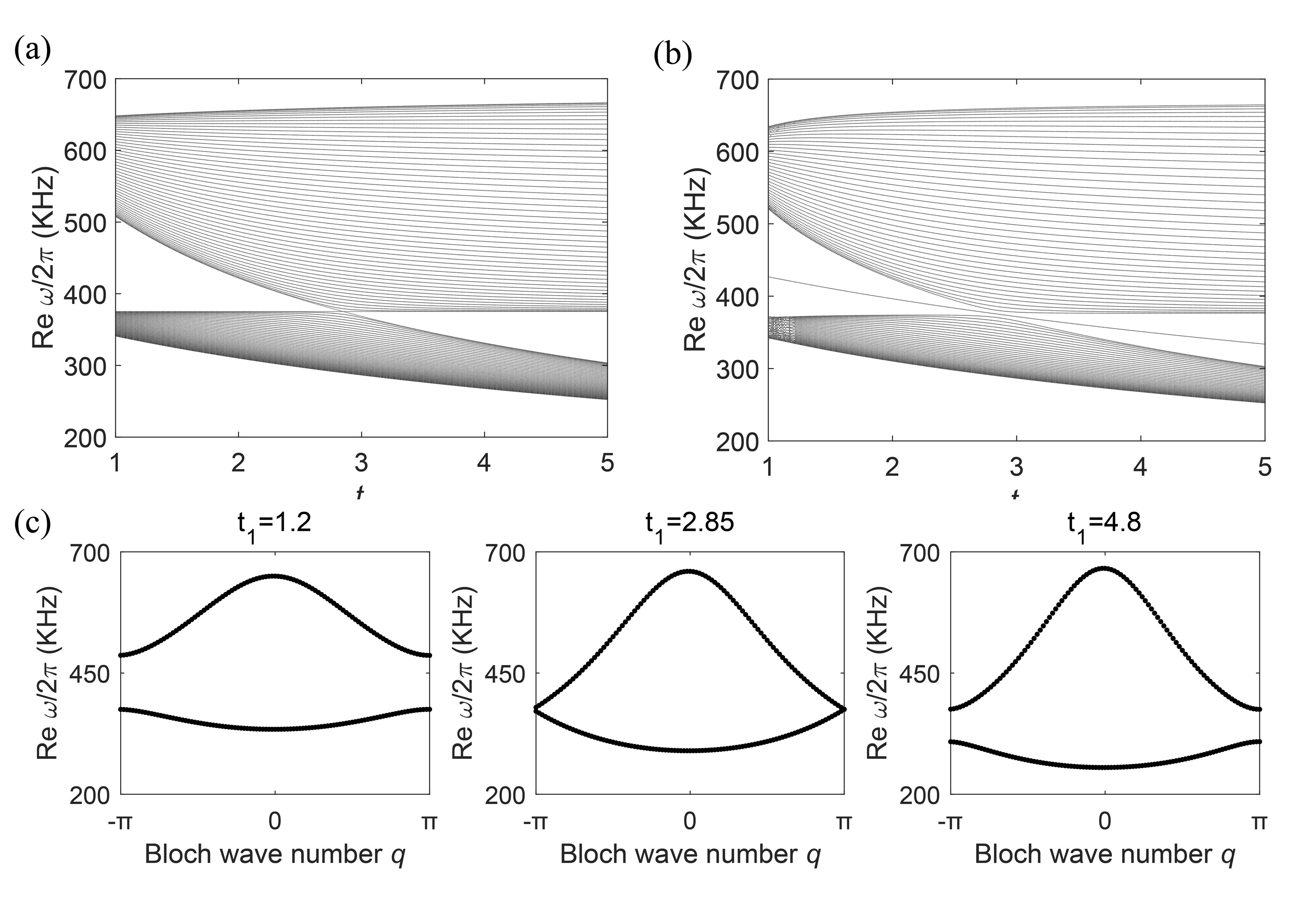


**Supplementary Figure S4. Band structures for the case** $\gamma_{1}\text{=-}\gamma_{2}\text{=}\gamma$, **with phase transition condition** $t_{1}\text{=}t_{2}$ **for both PBC and OBC. (a)** Bulk band structure as a function of *t_1_*. Each curve in **(a)** represents an eigenfrequency at certain Bloch momentum *q* in the first Brillouin zone (BZ). **(b)** Finite band structure as a function of *t_1_* of a finite circuit chain containing 40 unit cells. **(c)** Band structures of the bulk circuit at three different *t_1_* =1.2, 2.85, 4.8.


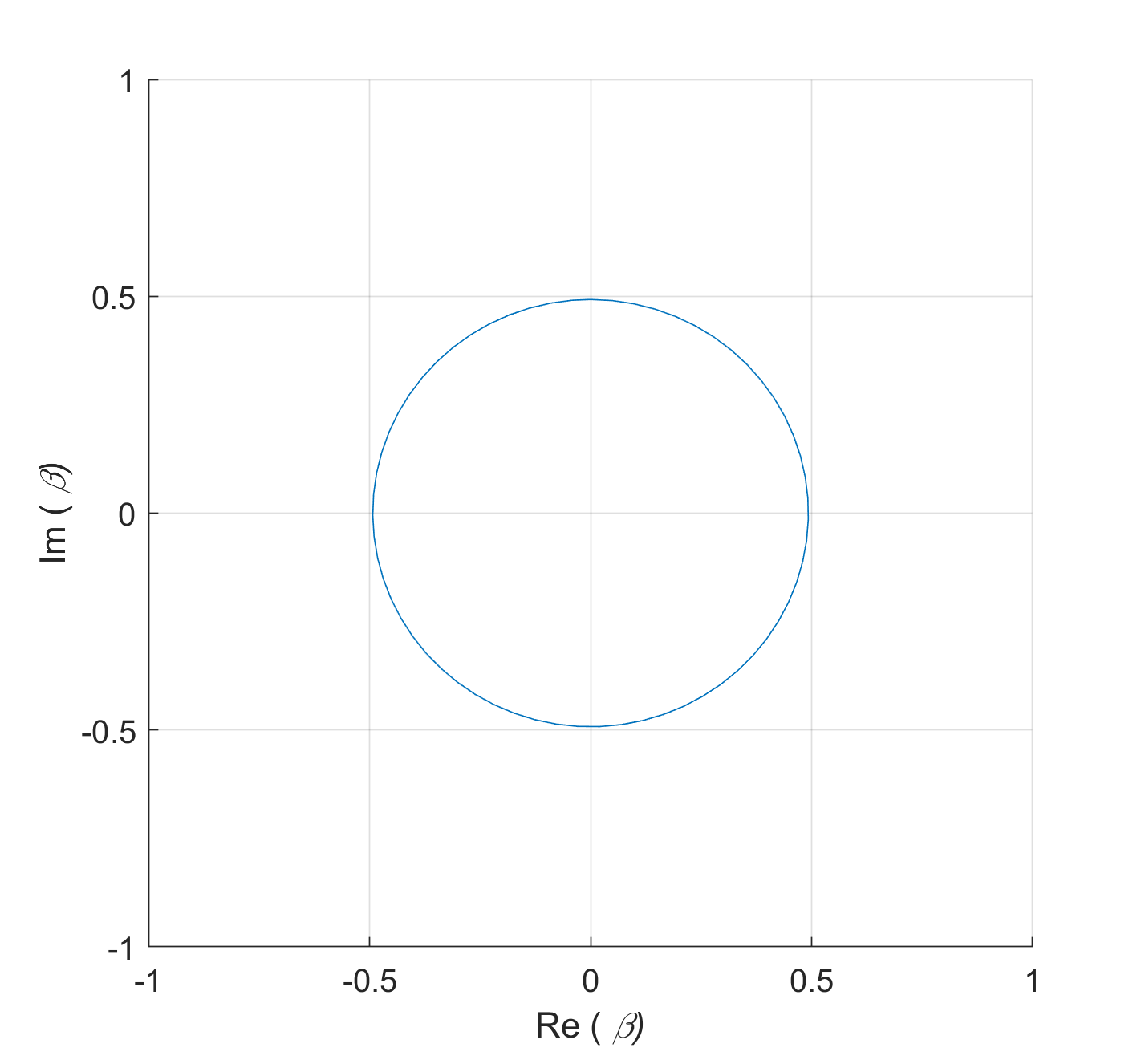


**Supplementary Figure S5. Plot of the generalized Brillouin zone with the circuit parameters used in the experiment. The localization factor *r=r_1_r_2_*in this case is 0.49.**


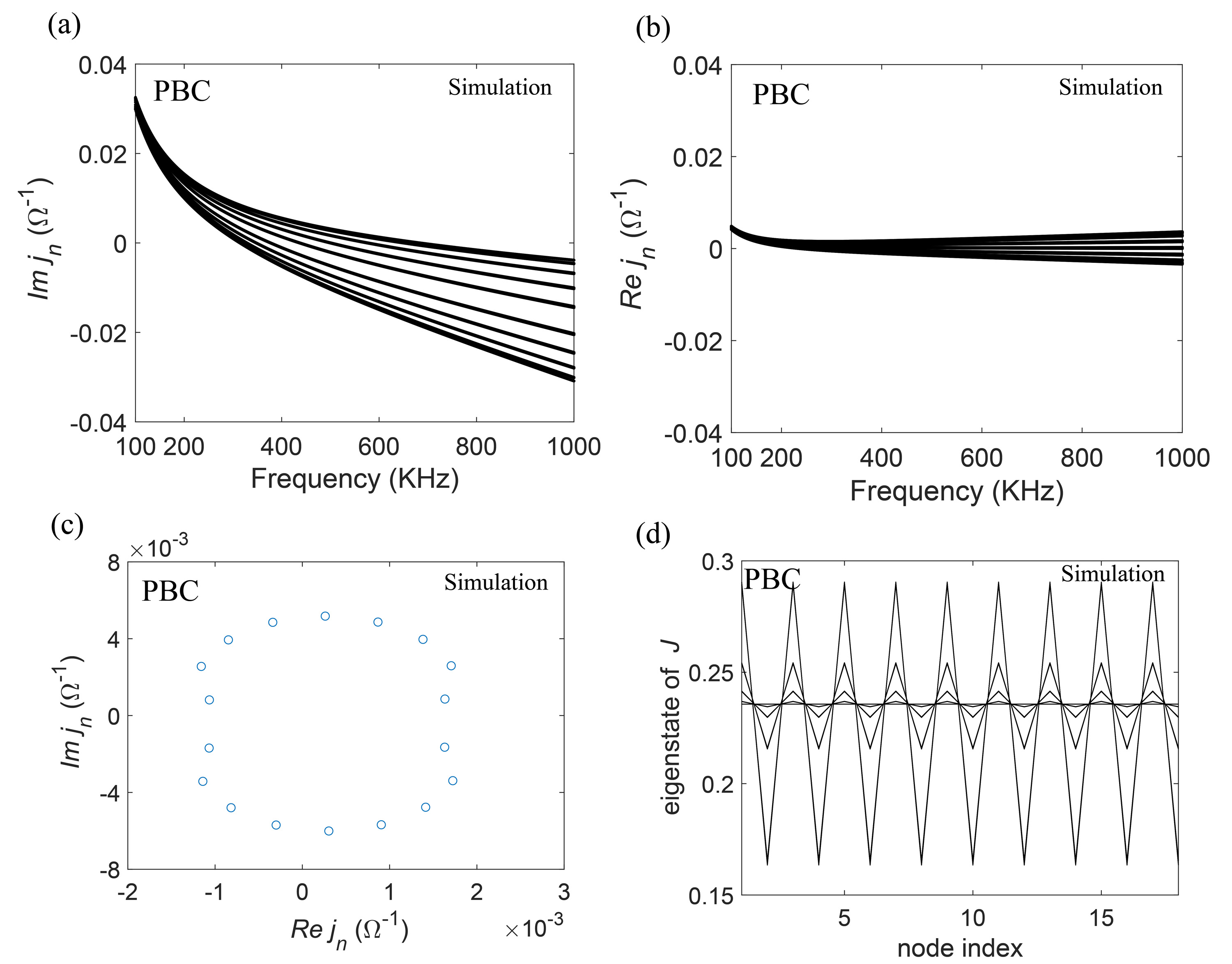


**Supplementary Figure S6. Numerical results for the eigenvalue and eigenstates of the finite circuit Laplacian for the finite circuit with PBC.** **(a,b)** Imaginary and real part of eigenvalues of the closed-loop circuit Laplacian, respectively. **(c)** Numerically calculated complex eigenvalues *j_n_*(*ω_0_*) of the circuit Laplacian at the mid gap frequency *ω_0_*. **(d)** Numerically calculated eigenstates of *J*(*ω_0_*) at the mid gap frequency *ω_0_*.


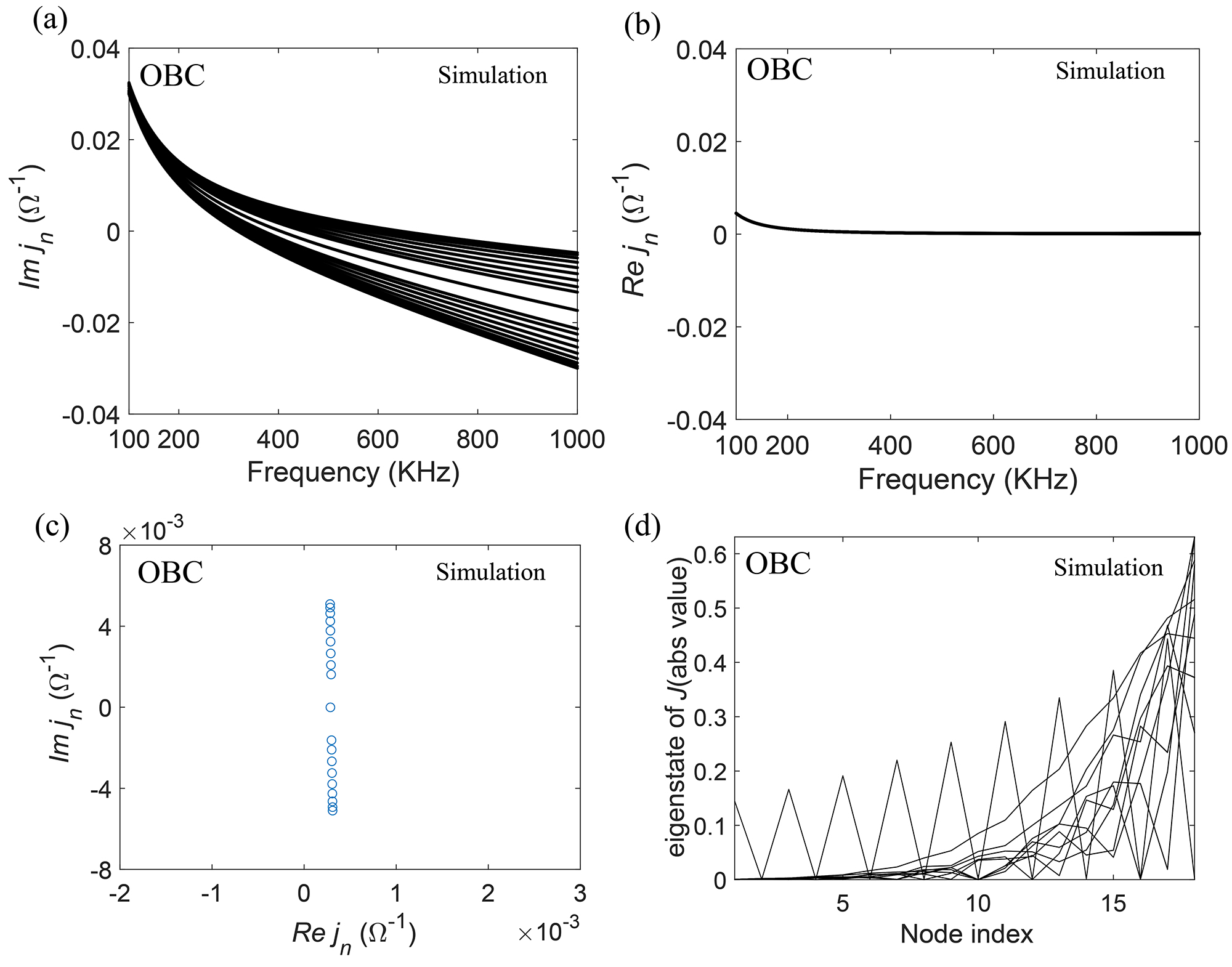


**Supplementary Figure S7. Numerical results for the eigenvalue and eigenstates of the finite circuit Laplacian for the finite circuit with OBC.** **(a,b)** Imaginary and real part of eigenvalues of the closed-loop circuit Laplacian, respectively. **(c)** Numerically calculated complex eigenvalues *j_n_*(*ω_0_*) of the circuit Laplacian at the mid gap frequency *ω_0_*. **(d)** Numerically calculated eigenstates of *J*(*ω_0_*) at the mid gap frequency *ω_0_*.

**Note S2. Effect of nonreciprocal coefficients**

Here, we examine the effect of on the non-Hermitian skin effect, namely, the direction and level of eigenstates localization of circuit Laplacian ***J****(ω_0_)* of an open circuit. For, all the eigenstates will localized at the left boundary of the circuit; for , all the eigenstates will localized at the right boundary of the circuit.

While for the cases , and , it is the product that determines the direction and level of non-Hermitian skin effect. For , all the eigenstates are localized at the left end of the chain; while for , all the eigenstates are localized at the right end of the chain. The level of localization is determined by the value of , namely, the larger is, the more eigenstates will concentrate at the circuit boundaries.

Supplementary Figure S6 shows the localization of eigenstates in the open circuit chain with and . The first example (Supplementary Figure S6 a and c) is with circuit parameter *C_0_*=470 pF, *C_1_*=470pF, *C_2_*=1000pF, *C_2_*=1000pF, *C_3_*=C_4_=680 pF, *L_0_*=47 μH, corresponding to$t_{1}\text{=1.72}$, $t_{2}\text{=2.85}$, $\gamma_{1}\text{=}-1.45$,$\gamma_{2}\text{=1.45}$, 1.56, 0.77, . The second example (Supplementary Figure S6 b and d) is with circuit parameter *C_0_*=470 pF, *C_1_*=470pF, *C_1_*=573pF, *C_2_*=752pF, *C_3_*=470pF, C_4_=1175 pF, *L_0_*=47 μH, corresponding to$t_{1}\text{=1.72}$, $t_{2}\text{=2.85}$, $\gamma_{1}\text{=}-1$,$\gamma_{2}\text{=2.5}$,$r_{1}\text{=1.35}$,$r_{2}\text{=0.62}$,.

As clearly shown in Supplementary Figure S6a and b, both cases exhibit almost the same band structure. However, as stated above, the eigenstates of the first case localize near the left boundary (Supplementary Figure S6c) while those of the second cases localize near the right boundary (Supplementary Figure S6d).


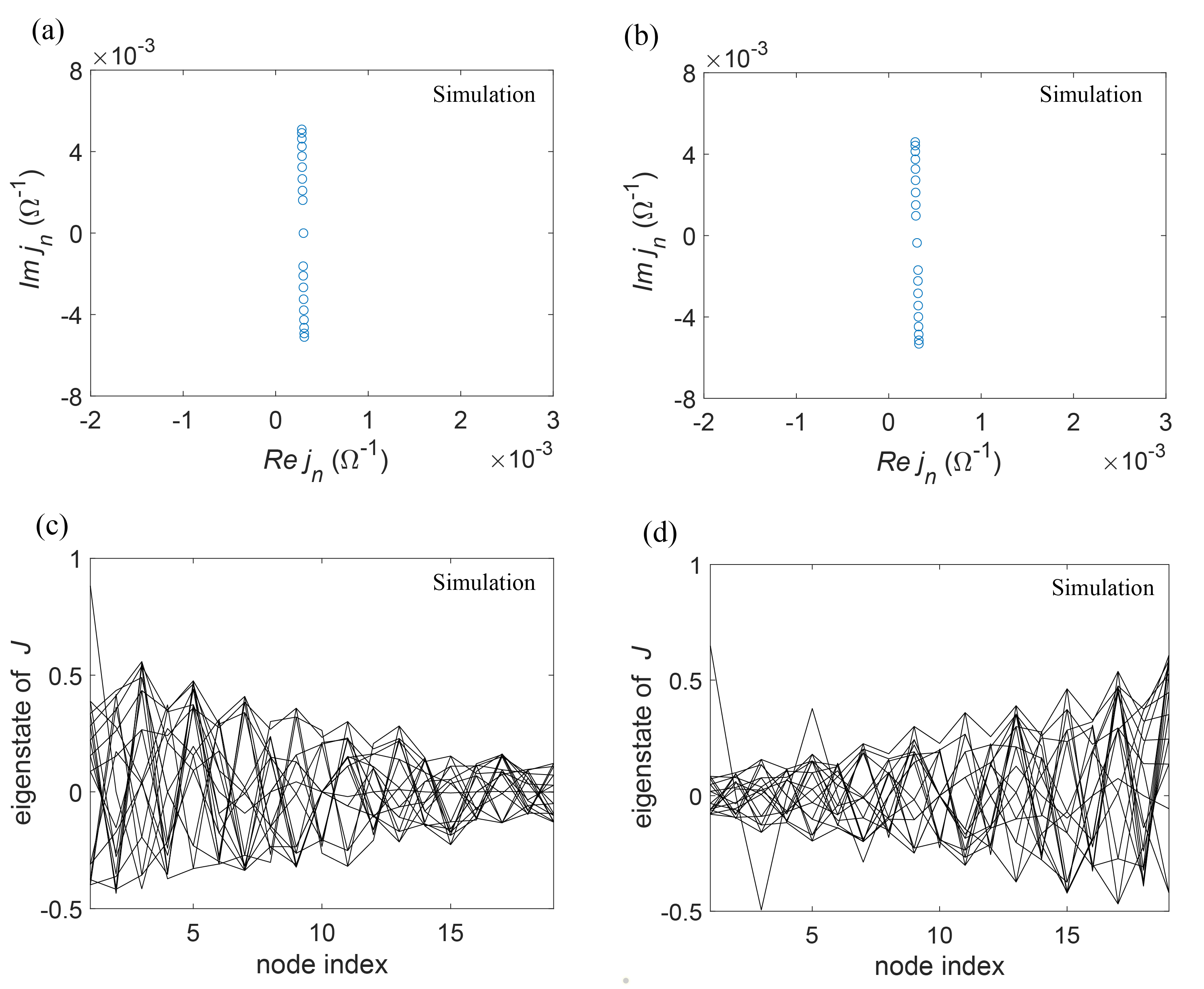


**Supplementary Figure S8. Band structures and localization of eigenstates in the open circuit chain with**  **and** . **(a,c)** Finite band structure and eigenstates of the circuit Laplacian for the circuit with , respectively. **(b,d)** Finite band structure and eigenstates of the circuit Laplacian for the circuit with , respectively.

**Reference:**

1. Takata, K., Notomi, M. Photonic Topological Insulating Phase Induced Solely by Gain and Loss. Phys. Rev. Lett. 121, 213902 (2018).
